# Comparative genomics of eight *Lactobacillus buchneri* strains isolated from food spoilage

**DOI:** 10.1186/s12864-019-6274-0

**Published:** 2019-11-27

**Authors:** Matthew A. Nethery, Emily DeCrescenzo Henriksen, Katheryne V. Daughtry, Suzanne D. Johanningsmeier, Rodolphe Barrangou

**Affiliations:** 1 Genomic Sciences Graduate Program, North Carolina State University, Raleigh, NC USA; 2 Department of Food, Bioprocessing & Nutrition Sciences, North Carolina State University, Raleigh, NC USA; 3 United States Department of Agriculture, Agricultural Research Service, Southeast Area, Food Science Research Unit, North Carolina State University, 322 Schaub Hall, Box 7624, Raleigh, NC 27695-7624 USA

**Keywords:** *Lactobacillus buchneri*, Comparative genomics, Lactic acid bacteria, CRISPR-Cas systems, Fermentation, Spoilage, Food microbiology

## Abstract

**Abstract:**

**Background:**

*Lactobacillus buchneri* is a lactic acid bacterium frequently associated with food bioprocessing and fermentation and has been found to be either beneficial or detrimental to industrial food processes depending on the application. The ability to metabolize lactic acid into acetic acid and 1,2-propandiol makes *L. buchneri* invaluable to the ensiling process, however, this metabolic activity leads to spoilage in other applications, and is especially damaging to the cucumber fermentation industry. This study aims to augment our genomic understanding of *L. buchneri* in order to make better use of the species in a wide range of applicable industrial settings.

**Results:**

Whole-genome sequencing (WGS) was performed on seven phenotypically diverse strains isolated from spoiled, fermented cucumber and the ATCC type strain for *L. buchneri*, ATCC 4005. Here, we present our findings from the comparison of eight newly-sequenced and assembled genomes against two publicly available closed reference genomes, *L. buchneri* CD034 and NRRL B-30929. Overall, we see ~ 50% of all coding sequences are conserved across these ten strains. When these coding sequences are clustered by functional description, the strains appear to be enriched in mobile genetic elements, namely transposons. All isolates harbor at least one CRISPR-Cas system, and many contain putative prophage regions, some of which are targeted by the host’s own DNA-encoded spacer sequences.

**Conclusions:**

Our findings provide new insights into the genomics of *L. buchneri* through whole genome sequencing and subsequent characterization of genomic features, building a platform for future studies and identifying elements for potential strain manipulation or engineering.

## Background

*Lactobacillus buchneri* is a lactic acid bacterium naturally found in varying ecological niches and is typically associated with food production and fermentation processes [1, 2]. This species has been isolated from a variety of environments, including fermented cucumber spoilage [3, 4], grass silage [5], a bioethanol production plant [6, 7], the human intestine and oral cavity [8, 9], cheese [10, 11], and in beer wort [12, 13]. It is a gram-positive, facultative anaerobe, and obligate heterofermenter producing lactic acid, acetic acid, ethanol, and carbon dioxide [14]. *L. buchneri* strains are morphologically and metabolically diverse, displaying an array of different colony phenotypes and can metabolize a wide range of carbohydrates [2]. Previous genomic characterization of *L. buchneri* CD034 revealed the presence of enzymes required to convert lactic acid to acetic acid and CO_2_ in the presence of oxygen, or 1,2-propanediol anaerobically, a unique metabolic feature protecting against acidification of the cytoplasm in the presence of large amounts of lactate [5]. This ability to convert lactic acid to acetic acid under both aerobic and anaerobic conditions makes *L. buchneri* useful in the aerobic stabilization of silage, effectively inhibiting spoilage organisms [15, 16]. While this feature is useful in certain bioprocessing environments, it can be detrimental to the cucumber fermentation process. *L. buchneri*’s metabolism of lactate leads to a rise in pH, enabling metabolic activity of less acid-resistant microbes, ultimately leading to the production of undesirable compounds that spoil the fermentation [4, 17].

It has been previously reported that lactic acid bacteria are highly adapted to specific ecological niches, and have small genomes compared to other bacteria as a consequence of a process called genome reduction, resulting in the maintenance of a minimal number of essential genes required for niche-specific survival [18]. Although the genome of *L. buchneri* is relatively small, it must retain the ability to quickly and continually evolve with its requisite environment, presumptively through horizontal gene transfer (HGT) of conjugative or mobilizable plasmids and transduction through bacteriophage (phage) infection [1]. Additionally, to survive and successfully propagate in a changing and highly-specific environment, the organism must balance the maintenance of robust defense systems against predatory phage and invasive plasmids with the genomic diversity created through the uptake of exogenous plasmids and other transmissible DNA elements. Alternatively, intra-species diversity can be generated through genomic duplication events propagated by the DNA-copying action of transposases [19, 20].

Although *L. buchneri* is reportedly diverse in isolation source, phenotype, and metabolic characterization, a mere 14 publicly-available draft genomes exist to date, only two of which are closed: NRRL B-30929 (NC_015428.1), isolated from an ethanol production plant [21], and CD034 (NC_018610.1), isolated from stable grass silage [5]. To elucidate genomic features, including the genetic flexibility of *L. buchneri*, we sequenced and assembled draft genomes of eight phenotypically distinct strains previously identified by Daughtry et al. [2] isolated from spoiled, fermented cucumber brine (LA1175D, LA1181, LA1184, LA1147), anaerobic reproduction of cucumber spoilage (LA1161B, LA1161C, LA1167), and tomato pulp (ATCC 4005). To generate an overview of the strains’ genomic similarity, we aligned the newly-assembled draft genomes with the two publicly-available closed reference genomes, NRRL B-30929 and CD034. Core- and pan-genomes across the eight isolates and two reference genomes were then determined, showing a marked level of genomic conservation. Annotated genes were each assigned a Clusters of Orthologous Groups (COG) designation for high-level functional assignment, indicating a significant number of non-conserved transposons and transposon-related sequences across the pan-genome.

Clustered regularly interspaced short palindromic repeat (CRISPR) and associated genes (*cas)* systems constitute the prokaryotic adaptive immune system and provide defense against phage and invasive plasmids through targeted nucleolytic cleavage [22–30]. CRISPR-Cas systems copy a short segment of DNA from the invading nucleic acid sequence and integrate it into the CRISPR locus as a template to prevent future attacks, called a spacer. This locus effectively serves as a “vaccination” record, storing infection events (spacers) chronologically [27, 31, 32]. Detailing and comparing these loci across strains provides insight into the ecological interplay between the isolates and invasive genetic elements, and can be used as a mechanism of strain genotyping [33–35]. CRISPR loci for the eight isolates and two reference strains were detected and repeats and spacers were identified and subsequently used to search for their genomic sequence of origin, called the protospacer. We show a surprising number of spacers target non-CRISPR regions of lactobacilli in areas containing putative prophage-related genes, as well as invasive plasmids.

Despite the wide range of phenotypes observed across these strains, we found that they share significant identity in terms of protein coding potential, as well as a high degree of similarity across their CRISPR-Cas systems, revealing identical repeat sequences and unique genotypic signatures constructed through the presence of shared ancestral spacers.

## Results

Whole-genome assembly was performed on each of the eight strains, revealing draft genome sizes between 2.49 Mb and 2.76 Mb (Table 1). The resulting number of assembled contigs > 1000 bp ranges from 20 to 128. Additionally, hybrid assembly using both short and long reads was performed on LA1184, resulting in 20 total contigs, 2 of which are closed plasmids: Contig 4 and Contig 6, with lengths of 53,573 bp, and 40,077 bp. All genomes share a similar GC content of ~ 44%, consistent with both reference strains NRRL B-30929 (44.4%) and CD034 (44.4%). Assembled genomes were then annotated to determine putative protein coding sequences, tRNAs, rRNAs, and CRISPR loci (Additional file 1: Table S1). The number of identified protein coding sequences ranges from 2377 to 2767.

**Table 1:** Whole-genome assembly statistics for each of the eight sequenced *Lactobacillus buchneri* isolates

Strain	Source	Genome Size (bp)	Contigs	N50 (bp)	Max Contig Size (bp)	GC%	Coverage	Sequencing Technology	Accession
ATCC 4005	Tomato pulp	2,493,071	67	64,594	174,839	44.3	48x	Illumina HiSeq	VFBO00000000
LA1147	Reduced NaCl fermented cucumber spoilage	2,608,988	128	36,444	93,082	44.1	46x	Illumina HiSeq	VFBV00000000
LA1161B	Anaerobic reproduction of commercial fermented cucumber spoilage	2,614,519	77	63,881	177,214	44	45x	Illumina HiSeq	VFBU00000000
LA1161C	Anaerobic reproduction of commercial fermented cucumber spoilage	2,561,573	60	78,131	198,440	44.2	47x	Illumina HiSeq	VFBT00000000
LA1167	Anaerobic reproduction of commercial fermented cucumber spoilage	2,613,434	73	61,779	136,582	44.1	56x	Illumina HiSeq	VFBS00000000
LA1175D	Reduced NaCl fermented cucumber spoilage	2,673,869	117	46,669	152,307	44.1	100x	Illumina HiSeq	VFBR00000000
LA1181	Reduced NaCl fermented cucumber spoilage	2,628,753	59	95,113	208,049	44	46x	Illumina HiSeq	VFBQ00000000
LA1184	Reduced NaCl fermented cucumber spoilage	2,761,236	20	2,348,394	2,348,394	44	500x	Illumina HiSeq + PacBio	VFBP00000000

**Fig. 1:**
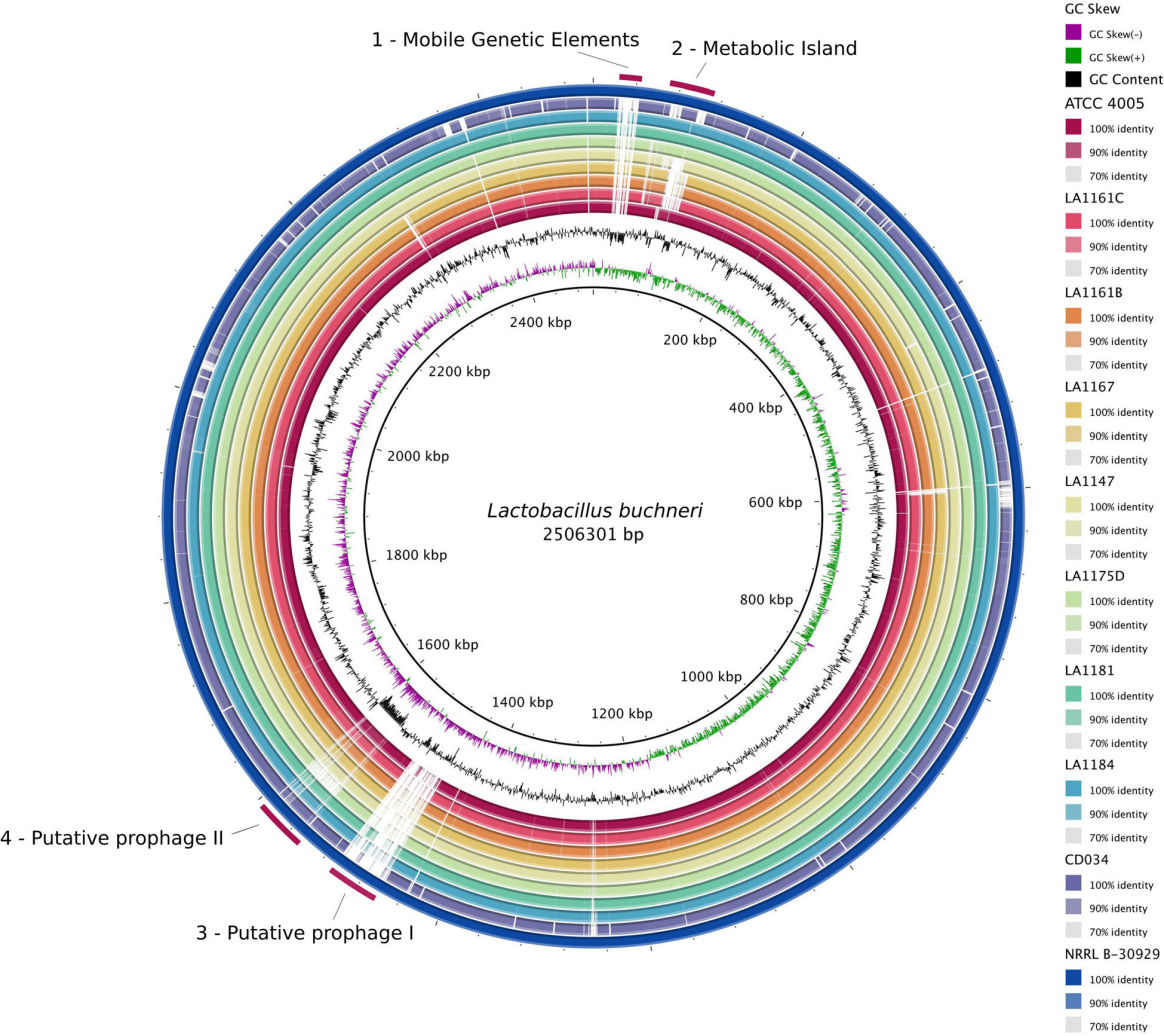
Genome-wide BLAST comparison of all isolates against reference strain NRRL B-30929. Four primary regions lacking significant coverage were identified: various mobile genetic elements, a metabolic island, and two putative prophages

Overall, when the predicted coding sequences of all strains were compared to the reference genome NRRL B-30929, we see a high percent identity within the BLAST identity range of 70 to 100% (Fig. 1). Notably, our group of isolates shares significantly more sequence identity with NRRL B-30929 than CD034. Upon further inspection, four primary gaps in coverage were identified through a low BLAST identity and noticeable decrease in GC content. The first gap in coverage (~ 21 kb) contains one integrase, one DDE transposase, two IS30 like transposases, and other regulatory proteins related to mobile genetic elements. The second identified region is ~ 40 kb long, containing 30 predicted open reading frames (ORFs). The majority of these sequences are predicted to code for various transporters, decarboxylases, and glycosylases. Most sequences encoded in this genomic island are found in NRRL B-30929, LA1184, LA1181, LA1175D, and ATCC 4005; however, their absence is observed in CD034, LA1147, LA1167, LA1161B, and LA1161C. The remaining two areas of sparse coverage each encapsulate a putative prophage. The putative prophage I region (~ 36.5 kb) appears to be unique to NRRL B-30929, whereas most of the coding sequences in the putative prophage II region (~ 38 kb) are common across all strains, with the exception of LA1175D.

**Fig. 2:**
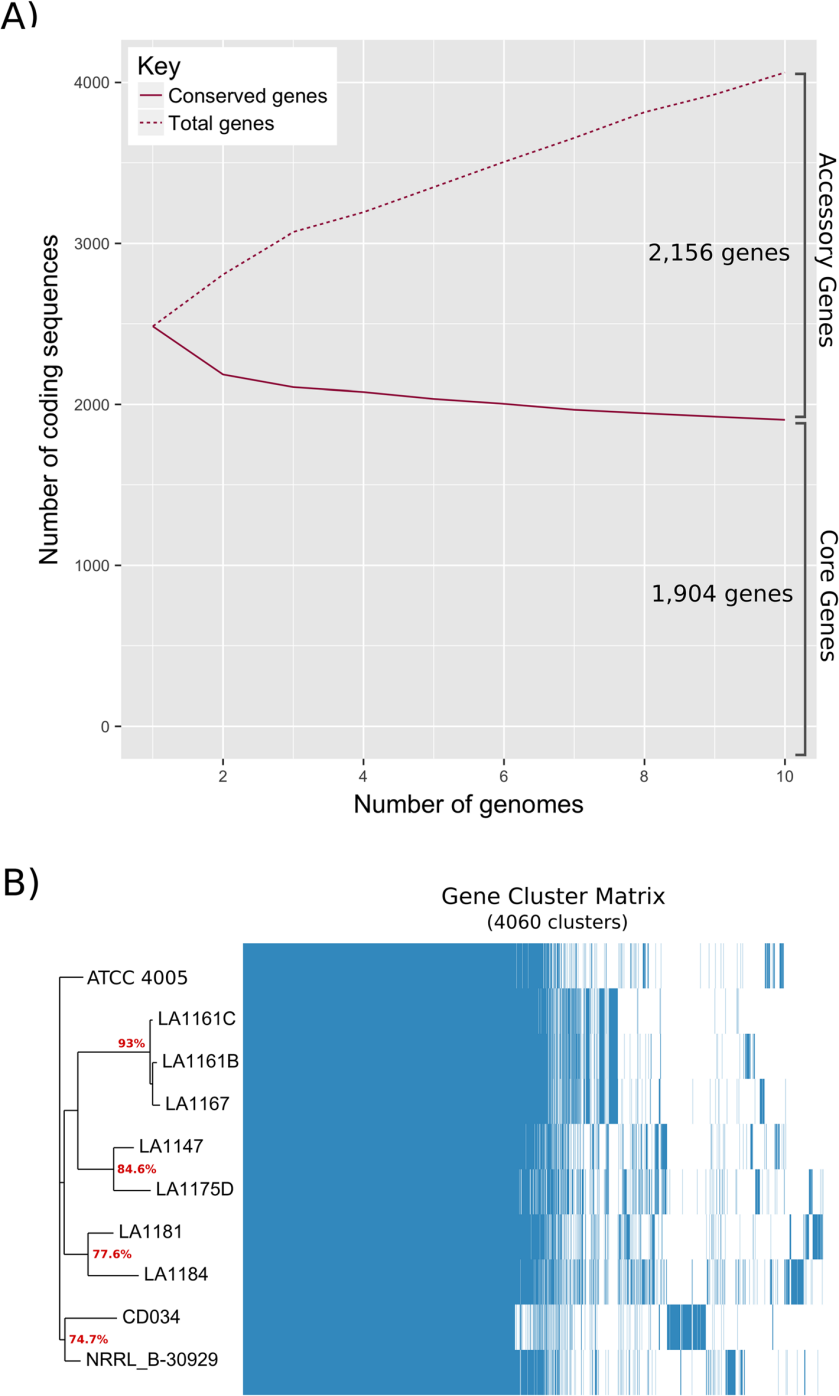
**a** Number of core genes across all strains plotted against number of accessory genes. **b** Core-genome based phylogenetic tree and gene cluster matrix comparing similar putative coding sequences

To characterize genomic conservation across the eight isolates and two reference strains, the overall coding potential of all ten strains was determined, called the pan-genome. Considering all protein coding genes identified across the pan-genome, we see slightly less than half of all genes conserved within a 95% BLASTP identity (Fig. 2A). Of the 4060 total coding sequences, 1904 were shared by all strains, comprising the core-genome. The non-core genes, termed accessory-genome, is composed of 2156 total coding sequences, likely contributing to the major phenotypic differences between strains as described by Daughtry et al. [2]. 1063 of these coding sequences are shared between 2 to 9 strains, while 1093 genes were found only in a single genome. When clustered by a gene presence/absence matrix, five distinct groups emerged (Fig. 2B). Group 1, comprised of LA1161B, LA1161C, and LA1167, displays the highest percent identity, sharing 93% of its coding sequences with only 153 sequences unique to an individual strain. Group 2, LA1147 and LA1175D, shares 84.6% of its coding sequences, having 419 genes unique to either strain, while group 3, LA1181 and LA1184, shares 77.6% of its coding sequences. The reference strains grouped together, showing 74.7% overall coding sequence identity, while the type strain ATCC 4005, isolated nearly 100 years ago, was the only member of its group.

**Fig. 3:**
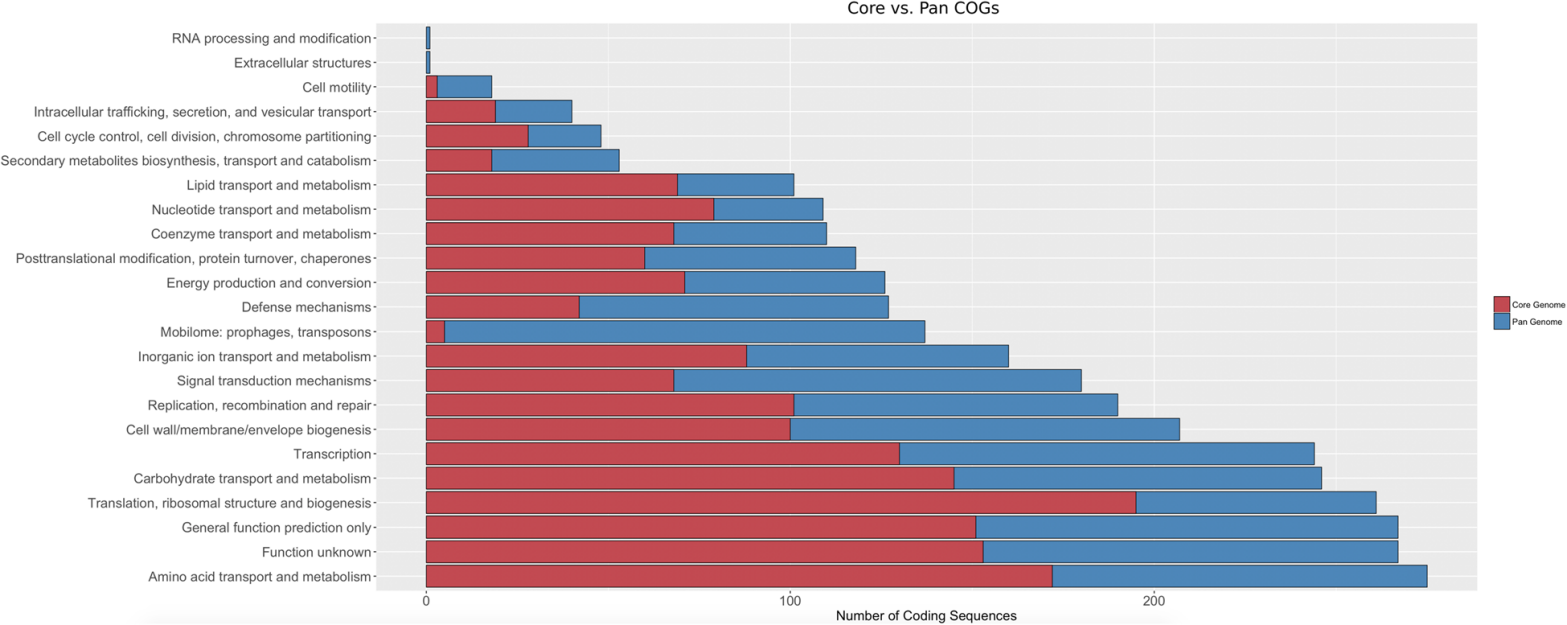
A comparison of functional COG groupings across the core- and pan-genomes

The core- and pan-genomes were annotated using the COG database [36] and assigned to functional groups (Fig. 3). As expected, the two largest core-genome categories contain coding sequences with functions related to translation, ribosomal structure, and biogenesis, as well as amino acid transport and metabolism. Interestingly, however, the third largest orthologous group, which encodes ~ 9% of the total core-genome, contains proteins of unknown function. Functional core-genome groups containing the least number of coding sequences belong to the ‘cell motility’, ‘mobilome’, and ‘secondary metabolite biosynthesis’ groups. Of note, the ‘mobilome: prophages, transposons’ group showed the lowest proportion between the number of core-genes vs the number of pan-genes, with only 5 sequences in the core-genome versus 137 in the pan-genome, illustrating exceptional diversity even across these highly related strains. Of these 137 pan-genome mobilome sequences, 76 belong to the transposons functional group or a closely related derivative category.

**Fig. 4:**
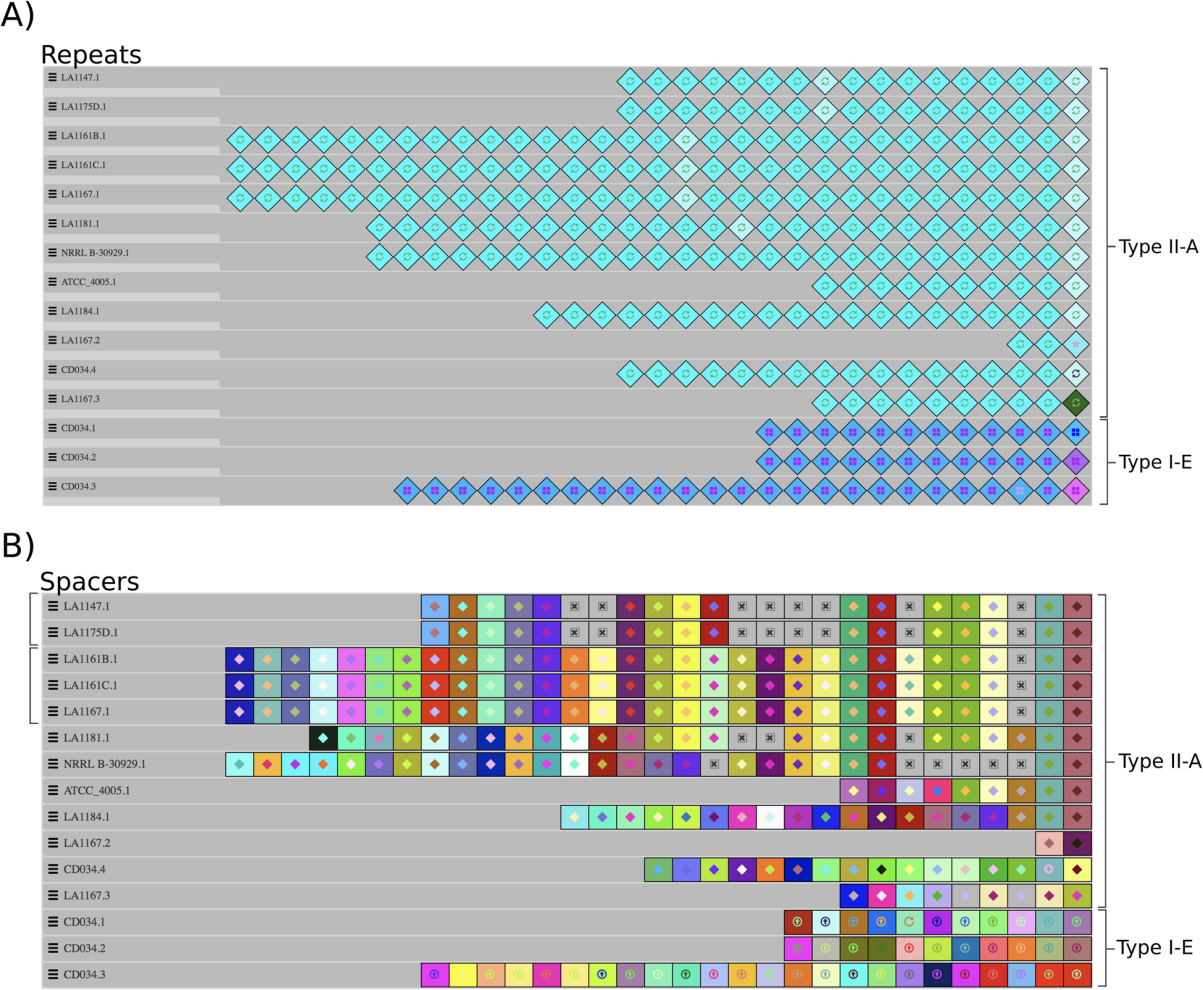
Visualization and alignment of repeat and spacer content for each detected CRISPR locus. Each diamond represents a CRISPR repeat, while each colored square represents a CRISPR spacer. Unique color combinations indicate distinct nucleotide compositions. Missing spacers are indicated by a gray “x” box. **a** Repeats are highly conserved across all isolates within CRISPR-Cas types II-A and I-E. **b** Some degree of shared evolutionary history is represented by the conservation of at least the first two ancestral spacers (on the right) in the type II-A spacer alignment

To bolster our understanding of the environmental interaction between these strains and invasive nucleic acids, we analyzed their CRISPR-Cas systems in detail. Location and identification of CRISPR-Cas systems were not hindered by the highly fragmented genome assemblies, and loci were successfully assigned a canonical type and subtype using standard tools and references (37, 38). Across the 10 strains analyzed, we found CRISPR-Cas systems belonging to both II-A and I-E canonical subtypes [37]. When grouped by repeat sequence and length, we see a type II-A system represented in all analyzed strains, as well as three type I-E loci unique to reference strain CD034 (Fig. 4A). All identified type II-A loci have a repeat length of 36 nt and a spacer length of 30 nt, with a range between 9 and 30 total spacers, with the exception of LA1167 CRISPR 2. Interestingly, LA1167 has a secondary type II-A CRISPR locus (CRISPR 2) with a full complement of *cas* genes ~ 12 kb downstream of its primary type II-A CRISPR 1 locus, although it contains only two spacers of unknown origin and three repeats. Two of the three repeats match the consensus repeat of CRISPR 1. The *csn2*, *cas2*, *cas1*, and *cas9* genes between LA1167 CRISPR 1 and LA1167 CRISPR 2 exhibit 88.74, 94, 93.77, and 82.43% amino acid identity, respectively. LA1167 also has a third locus, CRISPR 3, containing 10 repeat sequences but lacks any associated *cas* genes. Repeats at LA1167 CRISPR 3 match the repeat sequences of LA1167’s type II-A CRISPR 1 locus, indicating potential type II-A functionality.

Spacers from all CRISPR loci were extracted and aligned, positioning ancestral spacers on the right and more recent acquisition events on the left (Fig. 4B). With the exception of CD034, we see 100% identity across at least the first and second ancestral spacers from each strain’s type II-A CRISPR 1: a powerful confirmation of evolutionary homology [35]. Within this alignment, two groups with identical spacer sequences were easily identified. The first group contains LA1147 and LA1175D while the second contains LA1161B, LA1161C, and LA1167, consistent with the predicted core-genome based clades from the previous phylogenetic tree (Fig. 2B). While CD034 does have a type II-A locus, none of the identified spacers share significant identity with any type II-A spacer sequences from the other isolates.

**Fig. 5:**
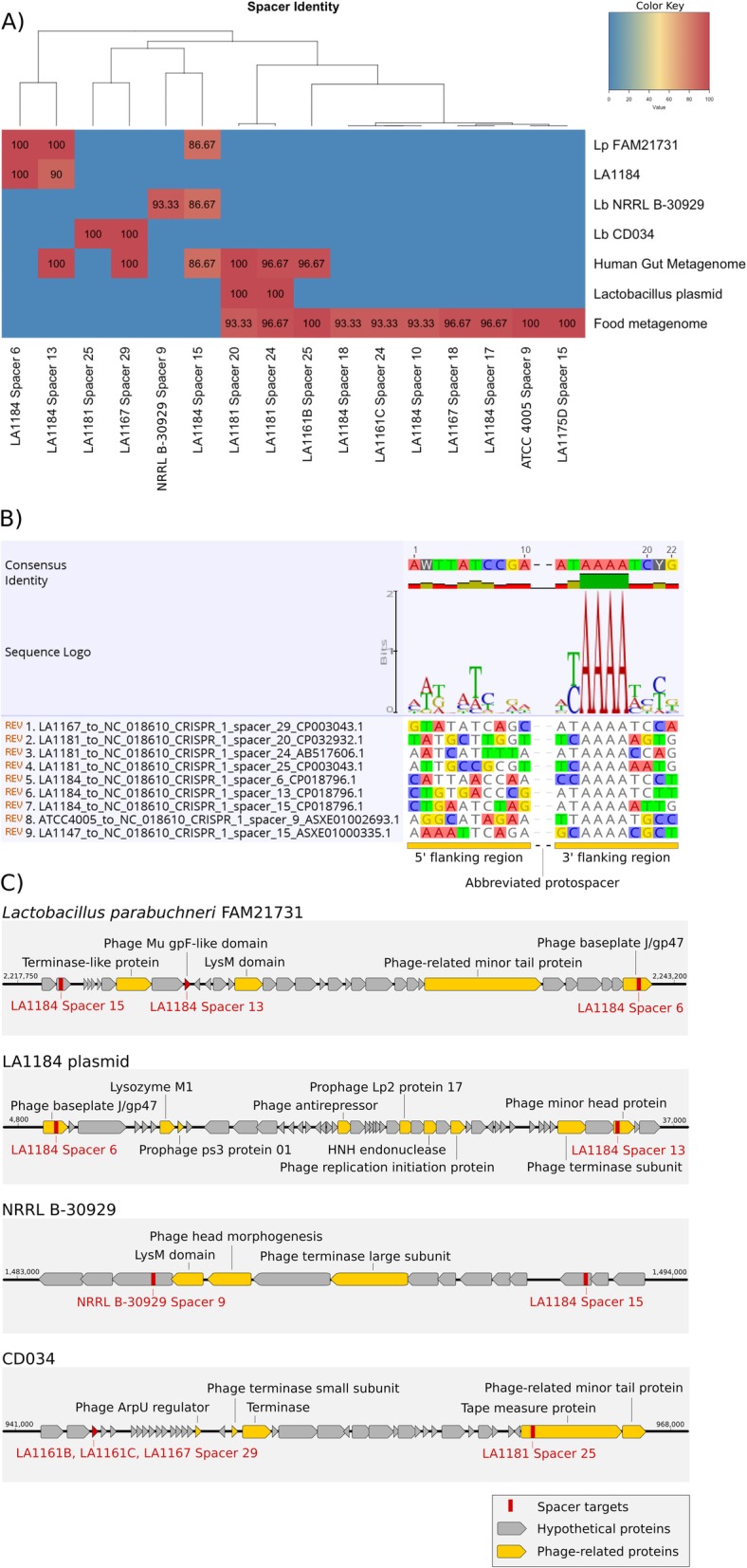
Characterization of type II-A spacers across *L. buchneri* isolates. **a** Genomic origins and sequence identity of 16 spacers. **b** The predicted PAM sequence for this type II-A CRISPR-Cas system, 5′ – AAAA – 3′, as identified by the alignment of 9 distinct protospacer-flanking sequences. **c** Protospacers of bacterial origin cluster together in putative prophage regions of the genome

Spacer origin was investigated with all available 273 spacer sequences via nucleotide BLAST searches [39]. A total of 16 protospacers were identified in the human gut metagenome, *Lactobacillus* plasmids, and various food metagenome samples, as well as within the genomes of *Lactobacillus parabuchneri* FAM21731, LA1184, NRRL B-30929, and CD034 (Fig. 5A). In all CRISPR-Cas systems except for type III, a conserved protospacer-adjacent motif (PAM) sequence is required for successful acquisition of new spacers and for interference [40–43]. The PAM sequence can be predicted through the alignment of flanking nucleotides among identified protospacer sequences [44, 45]. 9 distinct protospacers with > = 90% identity to corresponding spacers across 5 isolates were used in the analysis, yielding a predicted PAM of 5′ – AAAA – 3′, two nucleotides downstream of the protospacer (Fig. 5B). These results conform to a previously established PAM that was inferred from a wider selection of *L. buchneri* strains, including selected isolates used in this study, as well as several additional *L. buchneri* isolates not covered by this study [33].

The protospacers identified within four *L. buchneri* genomes were further explored. The three identified protospacers in the genome of *Lactobacillus parabuchneri* FAM21731 are clustered within a ~ 23 kb putative prophage region (Fig. 5C). LA1184 spacer 13 targets an uncharacterized conserved protein with a phage Mu gpF-like domain while LA1184 spacer 6 targets a phage baseplate J/gp47 family protein. The remaining spacer, LA1184 spacer 15, targets a hypothetical protein. Curiously, the two spacers found to match sequences in the LA1184 genome are self-targeting: they are encoded by LA1184’s own CRISPR 1 locus. Again, we see a protospacer match for LA1184 spacer 13, this time targeting a phage minor head protein with 95.92% similarity to the phage Mu gpF-like protein also targeted in *L. parabuchneri* FAM21731. The second self-targeting spacer, LA1184 spacer 6, targets a phage baseplate J/gp47 family protein in LA1184, identical to the targeted protein in *L. parabuchneri* FAM21731. Of the two protospacers found in the genome of NRRL B-30929, one is self-targeting: encoded by NRRL B-30929 spacer 9, and one is encoded by LA1184 spacer 15. NRRL B-30929 spacer 9 and LA1184 spacer 15 each target uncharacterized proteins within a ~ 8 kb region encoding several phage-related genes. The protospacers found in the genome of CD034 are matched by LA1181 spacer 25, which targets a phage tape measure protein, and LA1167 spacer 9 which targets a hypothetical protein ~ 20 kb upstream.

Due to the proposed lethal nature of self-targeting spacers, protospacer/spacer homology and associated PAM sequences for self-targeting spacers were investigated. LA1184 spacer 6 shows 100% identity with the matching protospacer sequence, but a single nucleotide polymorphism (SNP) exists in the PAM: AGAA. The protospacer matching LA1184 spacer 13 has the proper PAM (AAAA) but contains three consecutive SNPs on the 3′ end of the protospacer sequence in what is called the seed sequence [41]. Regarding NRRL B-30929 spacer 9, there are two SNPs in the middle of the protospacer sequence, as well as a single SNP in the PAM: ATAA.

Additional plasmid-based protospacer hits were also identified. LA1181 spacer 20 was found to target a type IV secretion system protein, TraC, in *Lactobacillus brevis* CD0817 plasmid pCD0817–1. LA1181 spacer 24 matches a sequence within a plasmid recombination enzyme mob 141 in *Lactobacillus plantarum* plasmid p141.

## Discussion

Given the expanse of colony morphologies and metabolic capabilities displayed by *L. buchneri*, as well as its prevalence in the food industry, there is a relatively low number of publicly available genome sequences. We sequenced and assembled draft genomes for eight phenotypically diverse strains of *L. buchneri*, a significant addition to the number of genomes available in the NCBI Genbank [46]. The range of assembled genome sizes, from 2.49 Mb to 2.76 Mb, are typical of the 1.8 to 3.3 Mb range reportedly found in lactic acid bacteria [18]. Hybrid genome assembly of LA1184 revealed 2 detectable plasmid sequences, consistent with multiple plasmids found in both reference strains NRRL B-30929 and CD034. Lactic acid bacteria are known to be highly specialized to their ecological niche, a hypothesis further supported by the presence of accessory plasmids that could quickly be acquired and transferred during times of rapid environmental change. We compared our draft genomes to the complete reference genomes of NRRL B-30929 and CD034 and note that in general, the eight strains share a higher percent identity with NRRL B-30929 than with CD034. Besides identifying two putative prophages, this comparison highlighted two genomic islands based on their divergent base compositions, a hallmark of HGT.

With only half of identified coding sequences conserved across all ten strains, there is a considerable level of genomic diversity represented in the accessory-genome. When each coding sequence is assigned to a COG, we see a large number of pan-genome sequences assigned to the ‘mobilome: prophages, transposons’ category versus the number in the core-genome, with more than half identified as transposons. This abundance and maintenance of diverse mobile elements suggest that transposons are likely an important genetic feature of *L. buchneri*. Interestingly, we see that ~ 9% of the core-genome is comprised of sequences with a currently unknown function, providing candidates for future functional studies. Additionally, we see that *L. buchneri* possesses a small set of putative secondary metabolite sequences. As a novel bacteriocin has been previously derived from *L. buchneri* [47], the potential for novel antimicrobials and other secondary metabolites should continue to be explored, given the variety of ecological niches occupied by this species and large number of coding sequences with no currently known function.

In all strains analyzed, a type II-A CRISPR-Cas system was found. In LA1167, we see a secondary type II-A locus (CRISPR 2) with only two spacers and a full set of *cas* genes. The presence of transposases on the 5′ and 3′ ends of this locus could signify a past duplication event in which the intact CRISPR 1 locus was copied locally. The small number of unique spacers and conserved repeat sequences in CRISPR 2, along with the differences in amino acid identity between the *cas* sequences of the two loci seem to indicate this locus remained functional post-duplication and has begun diverging from CRISPR 1 over evolutionary time. This view into a specific genetic duplication event can facilitate our understanding of transposon-mediated adaptation and the role it could play in bacteriophage and invasive plasmid defense as well as rapid niche-specific evolution. Across our eight strains and reference strain NRRL B-30929, we note the presence of at least two broadly shared ancestral spacers, evidence of a shared common ancestry and subsequent evolutionary divergence. The differences in spacer content displayed by CD034 and presence of three additional type I-E loci are additional signals of a more distant relationship to the eight strains than that of NRRL B-30929. Notably, the observation of spacers targeting similar or identical proteins in putative prophage regions across various *L. buchneri* genomes suggests multi-strain predation by a single phage or by several closely-related phages. Three spacers targeting the host’s own genome were identified. Assuming a fully functional CRISPR-Cas system, the expression of self-targeting spacers should result in host eradication, however, a variety of escape mechanisms have been observed [48, 49]. Two such escape mechanisms include mutating of the protospacer sequence or mutation of the PAM, which can lower binding efficiency of the CRISPR-Cas machinery to the target, reducing or inhibiting nucleic acid cleavage [41]. In the self-targeting spacers described here, we saw SNPs in the PAM sequence, SNPs in the protospacer, or both. SNPs in the PAM could prevent the initial binding of the Cas9 effector complex, while mismatches between the protospacer and RNA guide could prevent the conformational change required by the CRISPR-Cas effector complex for target cleavage [50]. Although individual phages may escape CRISPR-Cas targeting through random mutation, ultimately, they cannot avoid the random acquisition of spacers accrued by all cells of a bacterial population [51, 52]. An alternative phage defense mechanism against CRISPR-Cas targeting has been observed: the expression of CRISPR-Cas inhibitory proteins: anti-CRISPRs. These small phage-encoded proteins have been implicated in many cases in which self-targeting spacers have been observed, effectively inhibiting death of the host cell by prohibiting DNA cleavage by the CRISPR-Cas effector complex [53–55]. Anti-CRISPR proteins could play a role in the interaction between the host prophage regions and CRISPR-Cas machinery and is an attractive area for further study.

Indeed, food spoilage due to *L. buchneri* contamination and in some cases, potential pathogenicity, is of major concern to the food industry [3, 11]. The identity and activity of these prophages coupled with the knowledge of *L. buchneri*’s CRISPR-Cas systems could be exploited in the food biotechnology sector to treat and reduce contamination and spoilage by this organism, potentially preventing industrial loss and promoting robust bioprocessing. The CRISPR-Cas systems described here should undergo functional testing to explore their utility in engineering endogenous phage resistance into strains to protect starter cultures and promote the ensiling process. Additionally, in vivo characterization of detected prophages should be undertaken in an effort to develop novel tools for modulating this bacterial population and preventing food spoilage due to *L. buchneri* contamination in relevant industrial environments.

## Conclusions

The aim of this study was to increase the available body of knowledge on *Lactobacillus buchneri*, a microbe shown to have both beneficial and adverse effects in various industrial food settings. Whole-genome sequencing of 7 phenotypically diverse strains found in spoiled, fermented cucumber in concert with the ATCC type strain, ATCC 4005, were deposited in the NCBI Genbank, significantly increasing the number of publicly available *L. buchneri* strains. Further analyses revealed that these strains are highly enriched in mobile genetic elements, specifically transposons. A single type II-A CRISPR-Cas system was found in each of these strains, with the exception of LA1167, which was found to contain a second type II-A CRIPSR-Cas locus just downstream of the primary locus. Additionally, LA1184 and the reference strain NRRL B-30929 were found to encode CRISPR spacer sequences that target putative integrated prophage regions within their own genome and should be studied in further detail. By expanding the genomic sequences and characterization of this species we hope to set the stage for future studies and to provide actionable data for improved industrial bioprocessing.

## Methods

Cultures were obtained from the USDA-ARS Food Science Research Unit Culture Collection (Raleigh, NC). Genomic DNA was isolated using a kit (Qiagen DNeasy Blood and Tissue Kit) following the pretreatment protocol for Gram-positive bacteria. Resulting samples were sent to the High-Throughput Sequencing and Genotyping Unit of the Roy J. Carver Biotechnology Center at the University of Illinois at Urbana-Champaign for library preparation and sequencing. Sequencing was carried out on a HiSeq2500 using a TruSeq Rapid SBS sequencing kit with read lengths of 160 nucleotides. PacBio sequencing for LA1184 was performed by RTL Genomics (Texas, US). DNA from *L. buchneri* isolates was extracted for PacBio sequencing using Qiagen’s MagAttract HMW DNA Kit. Modifications to the protocol are as follows: 1) After the addition of P1 buffer and lysozyme, samples were incubated using a thermomixer at 37 °C at 900 rpm for ~ 1.5 h. 2) 100 μl AE was used to elute samples. Quality checking was performed using a dsDNA Broad Range DNA kit on a Qubit Fluorometer 3.0, as well as Fragment Analyzer by Advanced Analytical Technologies using the High Sensitivity Large Fragment 50 KB Analysis kit. Samples were subsequently processed through SMRTbell Library preparation using the following protocol: Preparing SMRTbell Libraries using PacBio Barcoded Adapters for Multiplex SMRT sequencing. The protocol was modified with the following: 1) Use equimolar pooled samples. 2) ~ 500 ng additional DNA was added per sample. 3) Use overnight ligation. 4) Use 12 μl of EB for final elution. Library quality was checked using dsDNA High Sensitivity DNA kit Qubit Fluorometer 3.0 and Fragment Analyzer using High Sensitivity large Fragment 50 KB Analysis kit. Library preparation for sequencing was completed using PacBio’s run protocol for diffusion loading, with the addition of a pre-extension time of 120 min and a final loading of 6 pM.

Adapter trimming, quality trimming, and filtering of raw fastq reads was performed in Geneious [56]. Reads were assembled using Geneious’ custom assembler with the following options: no trimming, save contigs > 1000 bp, don’t merge contigs when there is a variant with coverage over 6, allow gaps, ignore words repeated more than 200 times, use minimum overlap identity, use paired reads to improve assembly, and only use paired hits during assembly. Approximately 2.5% (~ 850,000 reads) of available short reads were extracted and used for assembly for all genomes except LA1175D (10% available reads used) and LA1184 (hybrid assembly). Using 2.5% of reads was determined using Assembly Likelihood Estimator [57], which indicated an undesired high number of short contigs as the percentage of reads used increased. The hybrid assembly of LA1184 used 2.5% of available HiSeq short reads, as well as PacBio long reads. Hybrid assembly with both short and long reads was performed using Unicycler [58] with the standard hybrid run options in normal mode, including SPAdes error correction [59]. To create scaffolds, contigs were aligned with both closed reference genomes, NRRL B-30929 and CD034, using Contiguator [60], which generated a single scaffold by concatenating contigs that aligned with the reference genomes. Gaps were inserted at concatenation sites with a series of 100 N’s. Unaligned contigs were added as additional sequence entries to the fasta file of each genome. Genomes were annotated using Rapid Annotation using Subsystem Technology (RAST) [61].

All vs NRRL B-30929 genome alignments were visualized using the BLAST Ring Image Generator (BRIG) [62], including a ring for each genome, as well as a ring for GC Content and GC Skew. A BLAST type of BLASTn [63] was used with the following options: upper identity threshold of 90% and a lower identity threshold of 70%, with a ring size of 30. Genetic features with low BLAST identity were identified through visual genomic inspection. The core- and pan-genomes were determined by first generating annotations using Prokka [64] with standard options that were then fed into Roary [65], using the flags -env and the standard threshold of 95% BLASTp [63] identity. The unique vs new genes graph was generated using the create_pan_genome_plots. R script distributed in the Roary package at https://github.com/sanger-pathogens/Roary/blob/master/bin/create_pan_genome_plots.R. The phylogenetic tree and gene presence/absence coverage were produced using the roary_plots.py script, also available in the public Roary distribution https://github.com/sanger-pathogens/Roary/tree/master/contrib/roary_plots. Core- and pan- genes were assigned a functional COG using PSI-BLAST [39] with the following flags: -show_gis -outfmt 7 -num_descriptions 1000 -num_alignments 1000 -dbsize 100,000,000 -comp_based_stats T -seg yes.

The COG database is publicly available for download here [36] https://www.ncbi.nlm.nih.gov/COG/. Core vs Pan COGs were visualized using RStudio [66] and ggplot2 [67].

CRISPR-Cas loci were identified, visualized, and aligned using CRISPRviz [38], and type was determined using the canonical definitions defined by Koonin et al. [68] after inspecting flanking *cas* genes and their corresponding annotations. Spacer identity was investigated using BLAST+ [39] against the nt, env_nt, and gss remote databases with the following flags: -task blastn-short -dust no -outfmt 5 -evalue 1e-5. The spacer identity heatmap was generated in RStudio using the gplots package available here: https://cran.r-project.org/web/packages/gplots/index.html. The PAM sequence was bioinformatically predicted using the procedure and CRISPRutils software package previously described by Nethery et al. [45] https://github.com/CRISPRlab/CRISPRutils

## Supplementary information

**Additional file 1: Table S1.** Number of protein coding sequences, tRNAs, rRNAs, CRISPR loci, and CRISPR repeats for each annotated isolate.

## Data Availability

The genomes generated and analyzed during the current study are available in the NCBI Genbank repository under the following accession numbers: VFBO00000000 (ATCC 4005), VFBP00000000 (LA1184), VFBQ00000000 (LA1181), VFBR00000000 (LA1175D), VFBS00000000 (LA1167), VFBT00000000 (LA1161C), VFBU00000000 (LA1161B), and VFBV00000000 (LA1147).

## Abbreviations

BRIG: BLAST Ring Image Generator
Cas: CRISPR-associated sequences
COG: Clusters of Orthologous Groups
CRISPR: clustered regularly interspaced short palindromic repeat
HGT: horizontal gene transfer
ORF: open reading frame
PAM: protospacer-adjacent motif
Phage: bacteriophage
RAST: Rapid Annotation using Subsystem Technology
SNP: single-nucleotide polymorphism
WGS: whole-genome sequencing
