# Supplementary material for: Comparative genomics of eight Lactobacillus buchneri strains isolated from food spoilage
Source: BMC Genomics. 2019 Nov 27;20:902. doi: 10.1186/s12864-019-6274-0 (PMC6881996; doi:10.1186/s12864-019-6274-0)
Supplement: Supplementary file 1 — Additional file 1: Table S1. Number of protein coding sequences, tRNAs, rRNAs, CRISPR loci, and CRISPR repeats for each annotated isolate. [file 12864_2019_6274_MOESM1_ESM.docx]

**Additional file**

Additional file 1: **Table S1.** Number of protein coding sequences, tRNAs, rRNAs, CRISPR loci, and CRISPR repeats for each annotated isolate.

| Strain | Protein Coding Sequences | tRNAs | rRNAs | CRISPR loci | Total Repeats |
| --- | --- | --- | --- | --- | --- |
| ATCC 4005 | 2,377 | 60 | 16 | 1 | 10 |
| LA1147 | 2,539 | 57 | 15 | 1 | 17 |
| LA1161B | 2,559 | 57 | 17 | 1 | 31 |
| LA1161C | 2,485 | 59 | 17 | 1 | 31 |
| LA1167 | 2,547 | 59 | 15 | 3 | 44 |
| LA1175D | 2,576 | 61 | 16 | 1 | 17 |
| LA1181 | 2,568 | 60 | 18 | 1 | 26 |
| LA1184 | 2,767 | 63 | 15 | 1 | 20 |
